# Choroidal Vascularity Index: An In-Depth Analysis of This Novel Optical Coherence Tomography Parameter

**DOI:** 10.3390/jcm9020595

**Published:** 2020-02-21

**Authors:** Claudio Iovino, Marco Pellegrini, Federico Bernabei, Enrico Borrelli, Riccardo Sacconi, Andrea Govetto, Aldo Vagge, Antonio Di Zazzo, Matteo Forlini, Lucia Finocchio, Adriano Carnevali, Giacinto Triolo, Giuseppe Giannaccare

**Affiliations:** 1 Department of Surgical Sciences, Eye Clinic, University of Cagliari, 09124 Cagliari, Italy; 2 Ophthalmology Unit, S. Orsola-Malpighi University Hospital, University of Bologna, 40138 Bologna, Italy; marco.pellegrini@hotmail.it (M.P.); federico.bernabei89@gmail.com (F.B.); 3 Department of Ophthalmology, Hospital San Raffaele, University Vita Salute San Raffaele, 20132 Milan, Italy; borrelli.enrico@yahoo.com (E.B.); ric.sacconi@gmail.com (R.S.); 4 Ophthalmology Department, Fatebenefratelli and Ophthalmic Hospital, ASST-Fatebenefratelli-Sacco, 63631 Milan, Italy; a.govetto@gmail.com (A.G.); giacinto.triolo@gmail.com (G.T.); 5 Vitreoretinal Division, Bristol Eye Hospital, University Hospitals Bristol NHS foundation trust, Bristol BS1 2LX, UK; 6 University Eye Clinic, DINOGMI, Polyclinic Hospital San Martino IRCCS, 16132 Genoa, Italy; aldo.vagge@gmail.com; 7 Department of Ophthalmology, University Campus Bio-Medico of Rome, 00128 Rome, Italy; antoniodizazzo@gmail.com; 8 Domus Nova Hospital, 48121 Ravenna, Italy; matteoforlini@gmail.com; 9 Department of Translational Surgery and Medicine, Ophthalmology, University of Florence, 50134 Careggi Florence, Italy; luciafinocchio@gmail.com; 10 Department of Ophthalmology, University “Magna Graecia”, 88100 Catanzaro, Italy; adrianocarnevali@live.it (A.C.); giuseppe.giannaccare@gmail.com (G.G.)

**Keywords:** choroidal vascularity index, choroidal imaging biomarkers, optical coherence tomography, retinal imaging

## Abstract

Remarkable improvements in optical coherence tomography (OCT) technology have resulted in highly sophisticated, noninvasive machines allowing detailed and advanced morphological evaluation of all retinal and choroidal layers. Postproduction semiautomated imaging analysis with dedicated public-domain software allows precise quantitative analysis of binarized OCT images. In this regard, the choroidal vascularity index (CVI) is emerging as a new imaging tool for the measurement and analysis of the choroidal vascular system by quantifying both luminal and stromal choroidal components. Numerous reports have been published so far regarding CVI and its potential applications in healthy eyes as well as in the evaluation and management of several chorioretinal diseases. Current literature suggests that CVI has a lesser variability and is influenced by fewer physiologic factors as compared to choroidal thickness. It can be considered a relatively stable parameter for evaluating the changes in the choroidal vasculature. In this review, the principles and the applications of this advanced imaging modality for studying and understanding the contributing role of choroid in retinal and optic nerve diseases are discussed. Potential advances that may allow the widespread adoption of this tool in the routine clinical practice are also presented.

## 1. Introduction

The choroid is a densely vascularized structure contributing to the majority of oxygen and other nutrients supply to the outer retina and the retinal pigment epithelium (RPE) [1]. It is one of the body tissues with the highest blood flow and plays a key role in the pathophysiology of many chorioretinal diseases [2].

Most of the anatomical knowledge of the choroid come from histological postmortem studies; although the advent of the optical coherence tomography (OCT) over the last decades has allowed its detailed and noninvasive in vivo examination.

Further recent advances in technology with the introduction of enhanced-depth imaging (EDI) OCT, swept-source (SS) OCT, enface OCT and OCT angiography (OCTA) have provided more in-depth analysis of the choroidal vasculature. Although choroidal thickness (CT) is considered a robust tool in clinical research, it reflects only the total choroidal vasculature with no distinctions between the two stromal and luminal vascular components [3,4,5].

In 2013, Branchini et al. firstly devised automated software based on MATLAB (Natick, MA: The Math Works Inc.) to calculate the area of dark and light pixels corresponding to luminal and stromal areas of the choroid (luminal choroidal area (LCA) and stromal choroidal area (SCA)) [6]. By means of this choroidal analysis, they demonstrated that the major components of the choroid are represented by the vascular lumen of the vessels.

Later in 2015, Sonoda and coworkers described a different technique to compute LCA and SCA using a Niblack binarization method of the EDI-OCT scans [7].

One year later, Agrawal and coauthors proposed a new quantitative parameter called choroidal vascularity index (CVI) as a novel OCT parameter for measuring the vasculature status of the choroid in healthy eyes [8] and for monitoring patients with panuveitis [9]. It was calculated as the ratio of LCA over TCA.

Through this new metric termed CVI, the authors evaluated the vascularity of the choroid confirming that in healthy eyes about two-thirds of the subfoveal choroid represented in a single cross sectional scan is vascular [8]. Numerous studies have been published so far regarding CVI and its potential applications in the evaluation and management of several disorders of the retina and the choroid [10].

In this review, the principles and the applications of this new OCT-based imaging modality are discussed, highlighting its potential benefits and future perspectives.

## 2. Technical Aspects

**Figure 1:**
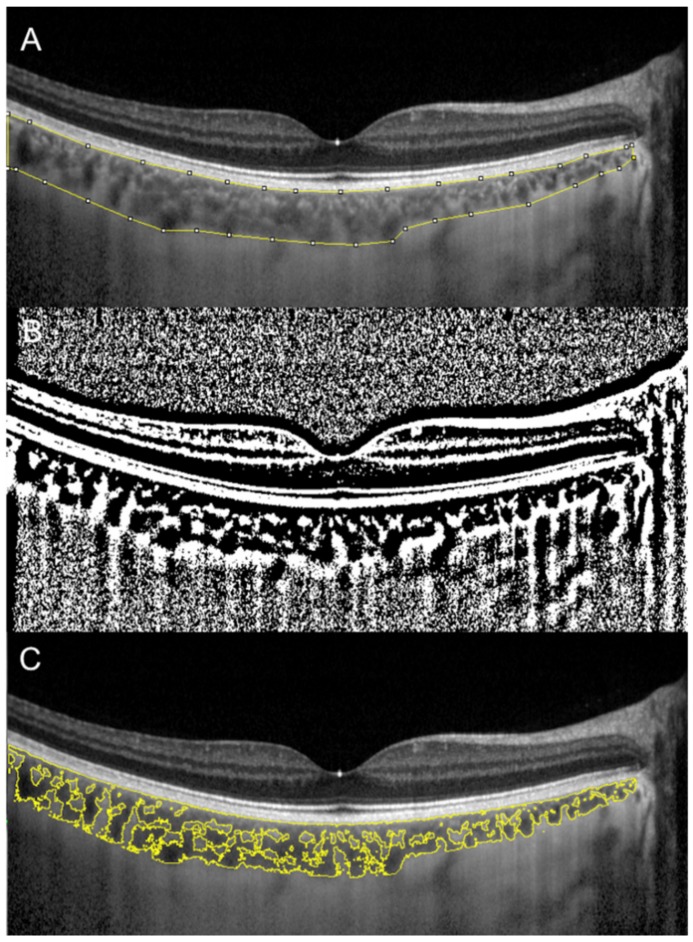
Choroidal vascularity index (CVI) calculation with binarization of enhanced-depth imaging (EDI) spectral domain optical coherence tomography (SD-OCT) image. Choroidal boundaries were traced to identify the total choroidal area (yellow lines) (**A**). The image was binarized using Niblack’s auto-local threshold (**B**). The color threshold tool was used to select the dark pixels, representing the luminal area (yellow lines) (**C**). The CVI is calculated dividing luminal area by total choroidal area.

Sonoda et al. firstly described a technique to evaluate subfoveal LCA and SCA by an image binarization process of the EDI spectral domain (SD)-OCT foveal scan using the free software ImageJ (National Institutes of Health, Bethesda, MD, USA) [7,11]. Briefly, the OCT image is opened in ImageJ, and the polygon tool is used to select a region of interest of 1500 µm wide, centered on the fovea. The upper boundary of the region of interest is traced along the choroidal–RPE junction and the lower boundary along the sclerochoroidal junction to identify the total choroidal area (TCA). Image brightness is adjusted on the base of the average value obtained from the LCA of three choroidal vessels selected using the oval selection tool. After conversion to an 8-bit image, Niblack’s autolocal threshold is applied to binarize the image and to demarcate the LCA and the SCA. The image is converted to a red, green and blue image, and the color threshold tool is used to select the dark pixels, representing the LCA. The TCA and LCA are finally measured (Figure 1). The SCA is then obtained by subtracting LCA from TCA. The ratio between LCA and TCA is calculated.

Several modifications have been further proposed to the first algorithm. In particular, Agrawal et al. renamed the ratio between LCA and TCA as CVI and made some methodological changes [9]. Firstly, in their technique, brightness is not adjusted as this would reduce the contrast between LCA and SCA and may affect the autolocal threshold process. Secondly, in order to ameliorate the visualization of the sclerochoroidal interface, the image binarization is performed prior to the area selection [8].

A study that compared the different algorithms showed poor agreement between the two methods [12]. Specifically, a higher CVI value was found using the method described by Agrawal et al. compared with the method described by Sonoda et al [7,9]. Nevertheless, it is not clear which of the two binarization algorithms is the most feasible in the evaluation of choroidal vascularity.

The CVI measurement has been further improved by means of contrast-enhanced imaging techniques that are able to increase the contrast between stromal and luminal areas, in agreement with true tissue regions. Furthermore, automated choroidal segmentation and binarization algorithms have been described, allowing a significant reduction in the time required for the evaluation [13,14]. However, to date there is no accessible published software for the automated calculation of CVI.

The comparison between CVI measurements on SD-OCT and SS-OCT scans was recently investigated and a good reliability between the measurements obtained with the two different wavelengths has been reported [15].

## 3. CVI in Healthy Subjects

Several studies aimed to establish normative values for CVI in healthy subjects [8,16,17]. Agarwal and coauthors investigated subfoveal CVI in a sample of 345 healthy eyes from subjects of the same ethnicity, with a mean age of 61 years [8]. Mean CVI calculated subfoveally with a width of 1500 µm was 65.61% ± 2.33%.

Interestingly, CVI presented a lower coefficient of variation compared with subfoveal CT (3.55 and 40.30, respectively). Moreover, in contrast to subfoveal CT that is affected by several physiological factors such as axial length, intraocular pressure (IOP), age and LCA, CVI value was affected only by subfoveal CT. In particular, a ticker subfoveal CT was significantly associated with a higher CVI [8].

Another recent study aimed to quantify vascular and structural macular variables in healthy eyes and to investigate correlations between these variables and age [16]. The authors reported no significant correlations of CVI with age or with retinal vascular parameters except for a negative correlation with foveal avascular zone area evaluated with OCTA [16].

In contrast with previous studies [8,16], Ruiz-Medrano and co-workers found CVI, along with TCA and LCA, to be significantly higher in the group with subjects under 18 years of age compared with the group including older subjects [17]. It can be hypothesized that LCA along with CVI decrease with ageing, while the SCA remains stable.

Topographical variation of CVI in healthy eyes was investigated [13,18,19]. Agrawal and coauthors analyzed choroidal vascularity comparing CVI obtained from (i) a single scan passing through the fovea, (ii) the mean values from scans in a 1000 microns area, based the inner circle of the Early Treatment Diabetic Retinopathy Study (ETDRS) grid and (iii) the mean value of macular scans from a raster of an area of 30 × 25 degrees [13]. Mean subfoveal CVI was 49.95% ± 4.84% and no significant differences were found compared with the other two scanning areas. Interestingly, these results highlight that in healthy subjects the evaluation of a single scan passing through the fovea is representative of the whole posterior pole choroidal vascularity.

These results were reinforced by a recent study evaluating the topographical variation of CVI in a three-dimensional macular area based on the ETDRS grid that reported no significant differences between different rings, subfields and quadrants of the grid [18].

The peripheral changes of CVI in the main four meridians of the fundus in normal subjects were also evaluated by means of SS-OCT [19]. The mean macular CVI was significantly smaller compared with superior, inferior, temporal and nasal quadrant. In this study, age was found to have a significant negative effect on CVI, whereas gender, refraction, IOP and axial length did not have any significant effect. A possible explanation for a lower CVI in the macular area may be related to the presence in this area of a thicker CT, a higher prevalence of medium vessels along with a thicker choriocapillaris compared to the peripheral ones that present a high rate of large vessels [19].

Diurnal variation of subfoveal and peripapillary CVI on SS-OCT scans performed at 2 h intervals from 09:00 to 17:00 was also investigated [20]. Subfoveal CVI along with subfoveal CT showed a significant diurnal change correlating with systolic blood pressure. Moreover, the mean peripapillary CVI in the temporal quadrant showed a significant diurnal variation as well [20].

Nagasato et al. evaluated the effect of water-drinking test on CVI and IOP [21]. The LCA, TCA, CVI and IOP significantly increased 30 min after the test and returned to baseline at 120 min. Moreover, changes of LA, TCA and CVI were significantly correlated with IOP fluctuations. It can be speculated that dilatations of the choroidal vessels after water-drinking test may lead to choroidal thickening with a subsequent IOP elevation [21].

All these findings in healthy subjects should be taken into account when evaluating the choroid in patients with retinal diseases.

## 4. CVI in Inherited Retinal Disorders

Choroidal vascular changes may contribute to the primary pathology of photoreceptor and RPE in inherited retinal disorders. CVI has been recently proposed as a novel OCT-based parameter to quantify structural changes in eyes with retinal dystrophies [22].

In a retrospective case control study, Wei et al. assessed CVI in patients affected by retinitis pigmentosa (RP) (*n* = 17), Stargardt disease (SD) (*n* = 4), cone–rod dystrophy (*n* = 3), Best disease (*n* = 1), and Bietti crystalline dystrophy (*n* = 1) [22].

They compared the retinal dystrophy group with a control group of 32 healthy subjects. All types of diseases were associated with a decreased CVI.

Tan and associates evaluated the structural changes in the choroid of patients with RP and showed a significant reduction in CVI as compared to normal eyes [23]. A lower CVI in RP supports the concept that choroidal vascular defects can influence the manifestation and progression of the disease. Therefore, CVI could be used as surrogate marker in monitoring and analyzing the progression of RP [23].

Iovino and coauthors studied the anatomical choroidal features associated with the presence of cystoid macular edema (CME) in eyes with RP [24]. A total of 159 eyes of 159 patients were enrolled and divided into two groups based on the presence (67 eyes) or absence (92 eyes) of CME. The choroid showed significantly decreased CVI in patients with CME associated with RP. These choroidal findings indicated the possible importance of choroidal hemodynamic alterations in the development of CME in RP [24].

Ratra et al. showed that CVI was significantly reduced in SD as compared to normal eyes and that the reduction of CVI was correlated with worsening of visual acuity [25]. They suggested that this novel parameter appears to be a more sensitive biomarker in detecting choroidal changes as compared to subfoveal CT.

## 5. CVI in Inflammatory Chorioretinal Disorders

The increased choroidal vascularity observed in posterior uveitis is interpreted as an indirect indicator of intraocular inflammation. In particular, Agrawal et al. evaluated CVI in patients with posterior uveitis and panuveitis, reporting an increased CVI in the affected eye, which significantly decreased after 3 months of follow-up [9].

An analogous result was obtained in patients with HLA-B27-associated anterior uveitis without clinically evident posterior segment inflammation [26]. Thus, choroidal vasodilation and vascular engorgement may be a nonspecific response to intraocular inflammation, possibly due to release of inflammatory and vasoactive cytokines. The normal CVI in patients with juvenile systemic lupus erythematosus without acute uveitis or a recent history of uveitis seems to strengthen this assumption [27].

**Figure 2:**
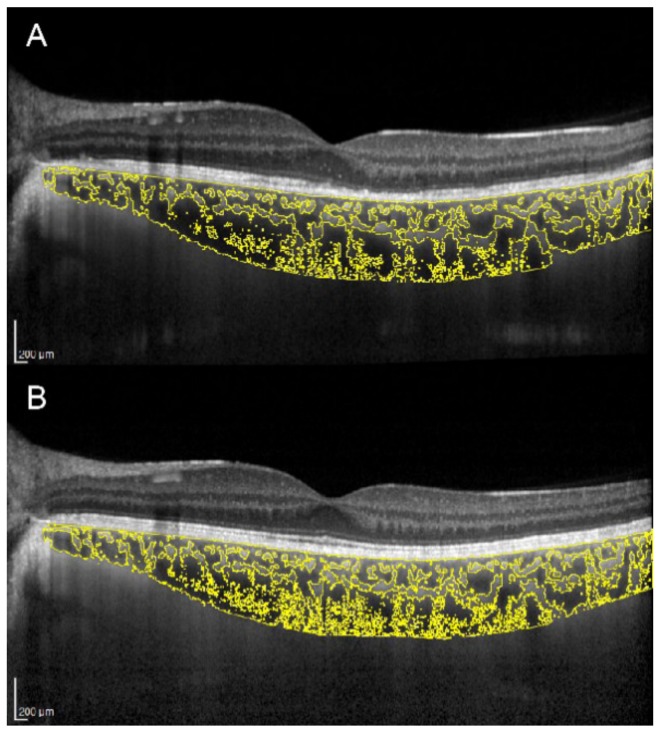
Choroidal vascularity index evaluation in a patient with multiple evanescent white dot syndrome. (**A**) In the acute stage, OCT shows ellipsoid zone disruption and a CVI of 69.3%. (**B**) In the healed stage, OCT shows normalization of the ellipsoid zone and a CVI decreased to 67.3%.

There is an ongoing debate as to whether the primary site of inflammation in multiple evanescent white dot syndrome (MEWDS) is the outer retina or the choriocapillaris. Increased CT and CVI were demonstrated in the acute stage of MEWDS, with subsequent reversal of choroidal changes along with outer retinal restoration and visual function recovery (Figure 2) [28]. This suggests an inflammatory involvement not only of the choriocapillaris, but also of medium and large choroidal vessels.

Nevertheless, it is still unclear if these choroidal changes play an active role in RPE/photoreceptor damage or are just a secondary effect of the inflammatory process occurring in the outer retina.

A decreased CVI was observed in other inflammatory disorders. A prospective study investigated choroidal alterations in patients with Behçet uveitis, reporting stromal expansion correlating with retinal vascular staining and leakage on fluorescein angiography [29].

The authors suggested that this finding could result from inflammatory cells accumulating predominantly in Sattler’s layer.

Significant choroidal changes were also reported in eyes with tubercular multifocal serpiginoid choroiditis (TB MSC), with increased CT and decreased CVI [30]. This is in agreement with the patchy hypocyanescence on indocyanine green angiography and the choriocapillaris flow void on OCTA observed in TB MSC. In the healed stage, patients developed inner choroidal atrophy with further CVI decrease [30].

A decreased CT with a normal CVI was observed in eyes with birdshot chorioretinopathy. This may indicate that the progressive choroidal thinning occurring in the disease affects both the stroma and choroidal vessels [31].

Four studies evaluated CVI in Vogt–Koyanagi–Harada (VKH) disease, reporting conflicting results [32,33,34,35]. A retrospective study on 19 eyes with VKH documented a higher CVI in the acute stage and reduction after treatment [32]. These results were confirmed by another prospective study [33]. The increased CVI was interpreted as an indirect indicator of stasis of blood flow within the choroid due to impaired choroidal circulation [32,33].

A study by Kawano et al. showed opposite results in VKH disease, with lower vascularity at baseline, which increased after 1 week of treatment [34]. Similarly, another study documented a lower CVI during active VKH, and subsequent increase after resolution. The authors hypothesized that the decreased CVI during active stage was due to choroidal stroma edema and infiltration by inflammatory cells [35]. These inconsistencies between studies may be due to methodological differences and technical difficulties in evaluating CVI in eyes with VKH, which often present a poorly visible choroidal–scleral junction due to a significant choroidal thickening.

Agrawal and coauthors reported a higher CVI in eyes with sympathetic ophthalmia, as a sign of a relatively larger extent of vasodilation compared to stromal exudation [36]. CVI may represent a potentially useful noninvasive tool to monitor disease activity, progression and response to therapy in patients with posterior uveitis.

## 6. CVI in Pachychoroid Disease Spectrum

Polypoidal choroidal vasculopathy (PCV), or aneurysmal type 1 neovascularization, is included in the pachychoroid disease spectrum [37,38,39]. In a large study including a total of 156 eyes from 156 patients (78 eyes with typical age-related macular degeneration (AMD) and 78 eyes with PCV), the authors demonstrated that PCV is characterized by a thicker baseline CT and greater LCA compared to those with AMD [40]. Based on these results, the CVI may be a useful metric for differentiating patients with neovascular AMD from those with PCV. Moreover, CVI was also demonstrated to be a useful parameter to differentiate two PCV subtypes: (i) PCV eyes with choroidal vascular hyperpermeability (higher CVI) and (ii) PCV eyes without choroidal hyperpermeability (lower CVI) [41].

Abnormalities in the choroidal vasculature including dilatation of choroidal vessels and leakage into the stromal space are well-known factors in the pathogenesis of central serous chorioretinopathy (CSC) [42]. Agrawal and coworkers evaluated CVI in the subfoveal choroidal area (1500 μ) of eyes with CSC and their fellow eyes [43]. The authors found an increased CVI in affected eyes compared with fellow ones. Moreover, contralateral eyes showed also a higher CVI in comparison with age-matched healthy subjects. Increased CVI suggests an increased choroidal vascularity in patients with acute CSC [43].

Retinal and choroidal changes were also investigated in steroid-associated CSC in comparison to idiopathic CSC [44]. Both acute and chronic forms were considered for the analysis. Steroid-induced CSC eyes showed a marginally higher CVI as compared to idiopathic CSC eyes. The authors speculated that the glucocorticoids may induce vascular dilation acting on mineralocorticoid receptors in the choroidal vascular endothelium, causing an increase of the CVI [44]. Choroidal vascularity has been analyzed subfoveally in CSC eyes with CNV and it was found to be significantly lower than non-neovascularized CSC eyes [45]. Choroidal changes, such as choroidal ischemia, can occur in eyes with lower CVI and this could be considered as a useful tool in evaluating the risk of CNV in CSC. Nevertheless, again it is difficult to assess if lower CVI is the cause of the effect of a CNV development in eyes with CSC.

Recently, CVI has been used to evaluate the treatment response in patients with CSC [46,47]. In particular, Rasheed et al. compared the effect of laser photocoagulation or observation on the choroidal vasculature in acute CSC [46]. They did not observe significant changes of treated eyes in comparison to observation, supporting the theory of no additional benefit of early laser photocoagulation in acute CSC [46].

**Figure 3:**
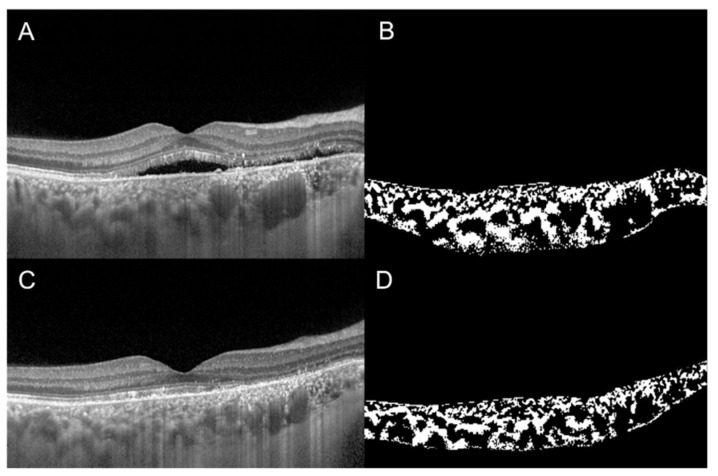
Choroidal vascularity index, calculated by the means of automated algorithm in a 56-year-old man with chronic central serous chorioretinopathy (CSC), before (**A**,**B**) and 3 months after (**C**,**D**) half-dose full-fluence photodynamic therapy, was 58.7% and 54.4%, respectively.

Additionally, CVI was evaluated to compare the effect of full-dose, half-dose (full fluence) and half-dose half-fluence photodynamic therapy (PDT) in CSC [47]. At 3-month follow-up in full-dose and half-dose (full fluence) group, CVI decreased and visual acuity improved significantly. The evaluation of CVI in a representative case of a 56-year-old man with chronic CSC, before and 3 months after half-dose full-fluence PDT, is shown in Figure 3. Conversely, in the half-dose half-fluence group it was significantly increased but no significant visual acuity improvement was recorded. The reduction of subfoveal CT after PDT was statistically significant in all three groups. The results of this study suggest that the effect of PDT on the half-dose half-fluence group was less clear than in the other groups [47].

Three-dimensional CVI was evaluated in treatment-naïve eyes with acute CSC by means of SS-OCT [48]. Volume data were obtained with a raster scan protocol covering an area 12 × 12 mm centered on the fovea. The three-dimensional CVI was defined as the ratio of the choroidal vascular luminal volume to the total choroidal volume, reflecting the volumetric choroidal vascular density. The eyes with CSC and the fellow eyes had significantly higher CVI values at the posterior pole compared to control eyes. Increased three-dimensional CVI confirms an increased choroidal vascularity in eyes with CSC and in their fellow eyes. CVI could represent a useful imaging biomarker for patients with CSC [48].

## 7. CVI in Age-Related Macular Degeneration

Alterations of the choroidal circulation are considered as factors potentially contributing to the development and evolution of AMD [49]. Although many factors have been implicated in the pathogenesis and progression of this disorder, there is strong evidence that AMD may be ultimately characterized by damage of the unit comprised by the photoreceptors, RPE, Bruch’s membrane and choroid [50,51,52,53,54,55,56].

Assuming this, evaluation of CVI provided additional insights into choroidal angio-architectural changes in AMD.

Previous studies employing structural OCT in early and intermediate AMD eyes have investigated changes in CVI [57,58]. The authors demonstrated that CVI is similar or slightly reduced in eyes with drusen, as compared with healthy eyes. More importantly, these studies showed that SCA was more represented in eyes with reticular pseudodrusen, suggesting a possible role of choroidal vascular depletion and fibrotic replacement in the pathogenesis and disease progression of this specific phenotype [57,58].

**Figure 4:**
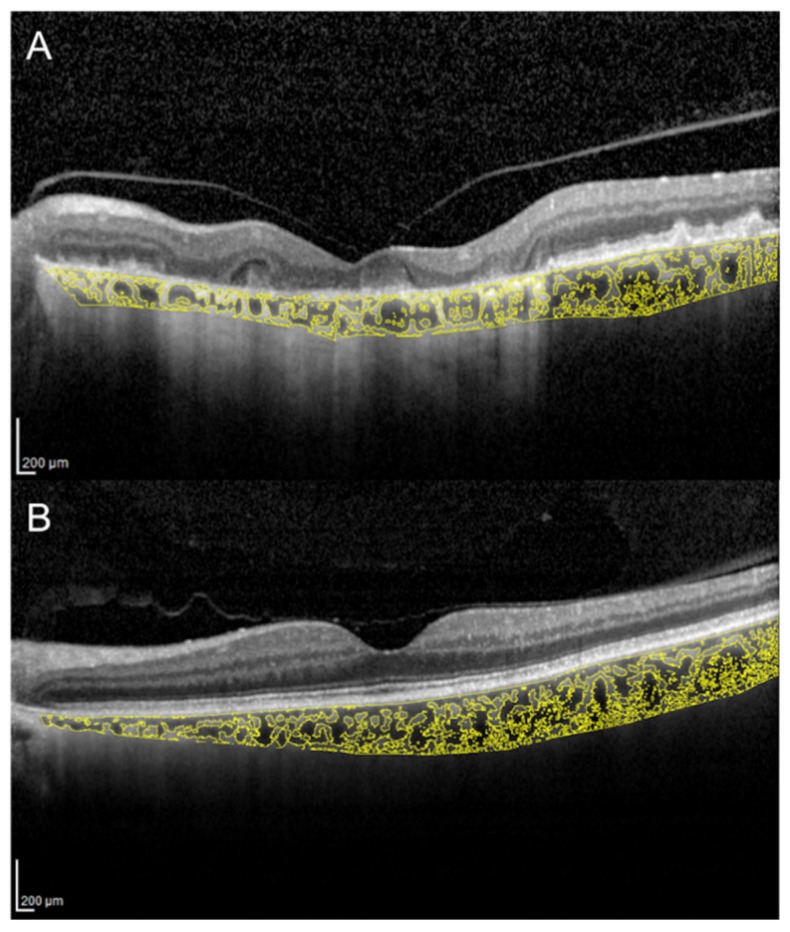
Choroidal vascularity index calculated with the OCT image binarization algorithm in a patient with geographic atrophy (**A**) and in an age-matched healthy subject (**B**) was 61.3% and 65.2%, respectively.

CVI was also investigated in eyes with geographic atrophy (GA) [59]. In the latter study, 34 patients with GA and 32 control subjects were retrospectively analyzed. The CVI was found to be reduced in patients with GA and it continued to reduce during the follow-up period (Figure 4). These results may suggest a stromal replacement of the choroidal vessels occurring in the setting of GA [59].

Invernizzi and colleagues performed a longitudinal analysis to correlate changes in CT and CVI with disease activity in eyes with neovascular AMD [60].

Eyes with a diagnosis of nonexudative AMD at the first examination who presented signs of exudation during the subsequent visit were enrolled. They demonstrated that CT and CVI significantly increased with active disease in neovascular AMD eyes. Of note, only type 1 lesions demonstrated statistically significant modifications in CVI from the inactive to the active form of the disease. The authors argued that these results may be secondary to the fact that type 1 CNV is located beneath the RPE, which may increase the diffusion of inflammatory mediators within the choroid [60].

In the attempt to explore the vascular compliance in eyes with AMD, Yiu et al. evaluated the choroidal response to a single dose of oral sildenafil citrate [61]. After sildenafil, CT significantly increased, and this was primarily attributed to the expansion of the SCA. The choroidal response was less pronounced in older patients, but it was not affected by the presence of exudative or nonexudative AMD [61].

## 8. CVI in Myopia

A precise, reproducible, noninvasive tool like CVI may be critical in high myopia (defined as spherical equivalent (SE) of 6.0 diopters or worse), as in such eyes the choroid can be extremely thin, as reported in numerous studies [62,63]. In myopic eyes, subtle changes in CT and volume may be difficult to quantify with qualitative, observer-dependent subjective analysis.

In high myopia, several hypotheses have been made to explain the pathophysiology of choroidal thinning, mainly focusing on vascular and mechanical abnormalities [64]. Vascular abnormalities include narrowing and loss of large choroidal vessels, occlusion of choriocapillaris and ischemia, whereas mechanical abnormalities include stretching of the sclera and choroidal stroma [64].

To define the pathophysiology of choroidal thinning in high myopia is important, as it may help to refine more efficient therapeutic strategies in such patients. The CVI may help to address such important issue.

In the published literature, few reports studied choroidal vasculature in highly myopic eyes using the CVI [64,65,66].

Alshareef and co-authors analyzed retrospectively the subfoveal CVI of 30 myopic eyes versus a nonmyopic control group, and found significant differences in the SCA, which was smaller in myopic eyes [65]. Conversely, no differences were encountered in the LCA [65]. In a prospective study, changes in CVI were examined in myopic eyes with and without myopic neovascular membranes [64]. Although, similarly to AMD, CT decreased significantly after anti-VEGF [67], there were no changes in CVI between eyes with and without myopic neovascular membranes. This may suggest that the choroidal thinning seen after anti-VEGF injections is due to mechanical rather than vascular factors [64].

However, in a recent, larger cross-sectional report that included more than 500 myopic eyes, Gupta and coworkers suggested that myopic choroidal thinning may involve both vascular and mechanical pathways, in contrast with the findings from Alshareef and Yan Ng [66].

To date, the usefulness of the CVI in high myopia is still controversial, although promising. Due to the low number of high-quality reports on this matter, the scientific evidence remains weak.

The CVI may have the ability to distinguish between stromal and vascular component in high-myopia-associated choroidal thinning. However, further larger, prospective and controlled reports may help to clarify the role of CVI in the clinical management of highly myopic eyes.

## 9. CVI in Diabetic Retinopathy

The choroid plays an important role in the pathogenesis of diabetic retinopathy (DR). Several studies attempted to compare the subfoveal CT of patients with DR and healthy subjects, with controversial results [68,69,70]. According to a recent review analyzing the currently available literature on diabetic choroidopathy, it is unclear if choroidal changes in patients with diabetes are predictive, modulatory, causative or independent factors for DR [71]. CVI has been proposed as a novel OCT parameter for disease monitoring in DR [72]. The authors found a decreased CVI with no corresponding change in CT in patients affected by diabetes mellitus.

Gupta and associates analyzed the structural choroidal changes occurring in patients with DR, concluding that subfoveal CT and CVI are dynamic parameters affected by diabetic macular edema [73]. Unlike CVI, subfoveal CT is also affected by ocular and systemic factors like edema and hypertension [73].

Interestingly, CVI was also measured in conjunction with DR stage [74]. Patients with proliferative DR exhibited a significant lower CVI value than those with mild or moderate non-proliferative DR and healthy subjects. In a multivariate regression analysis, thicker subfoveal choroid and thinner central retina were significantly associated with higher CVI values. The authors suggested that changes in choroidal vasculature, evaluated with CVI, could be the primary events in diabetes even where there are no signs of DR [74].

## 10. CVI in Glaucoma

As the choroid can be influenced by many physiological variables, a marked disparity exists on the impact of glaucoma on CT analysis [75]. In fact, the measurement of thickness is only partially representative of the choroid’s overall structural change.

Based on this consideration, CVI may help to elucidate the role of choroidal vasculature in the development and progression of several retinal and optic nerve diseases, including glaucoma.

The CVI was found to be significantly reduced in open angle glaucoma (OAG) and pre-perimetric glaucoma (PPG) eyes compared to healthy eyes [76]. The same study also showed that OAG and PPG eyes had lower LCA than healthy eyes. Based on these findings, the authors speculated that choroidal ischemia may influence the pathogenesis of glaucoma or may be a result of glaucomatous damage [76].

Previous studies found that reduced volume of juxtapapillary choroid was associated with β-zone parapapillary atrophy (βPPA) in glaucomatous eyes [77,78].

Since the introduction of OCTA, parapapillary deep-layer microvasculature dropout (MvD_P) was observed by means of complete dropout of the choriocapillaris or microvasculature within the sclera in βPPA [77,78]. MvD_P has been reported to be associated with glaucomatous visual field (VF) damage and focal lamina cribrosa defect [77,78].

While evaluating the choroidal microvasculature within the βPPA with OCTA is relatively straightforward, the same is not true for the choriocapillaris outside the βPPA, due to projection artifacts from the highly reflective deep vasculature within the RPE.

In this view, the CVI is an OCT parameter that may be representative of choroidal microvasculature outside the βPPA and therefore may add useful information about the role of this anatomical site in the pathogenesis of glaucoma.

Park et al. recently reported that reduced CVI outside βPPA is associated and topographically related to the impaired deep-layer microvasculature within βPPA in glaucomatous eyes [79]. Interestingly, in this study CVI did not show any significant relationship with retinal nerve fiber layer thickness, VF mean deviation or pattern standard deviation [79]. However, further studies with large numbers of study subjects are required to better elucidate the relationships between glaucoma severity, CT, CVI and MvD_P.

## 11. CVI in Ocular Surgery

Few reports used the CVI to analyze choroidal changes after cataract and macular surgery, and none of them has identified the CVI as an effective biomarker to predict postoperative functional or anatomical results [80,81,82]. The identification of reliable prognostic biomarkers is critical to improve the ability of clinicians to predict postoperative outcomes, especially in macular surgery [80].

Despite the lack of a true, practical use of CVI for the cataract or vitreoretinal surgeon, CVI analysis may be useful to better understand the pathophysiology of postoperative choroidal volume changes.

A significant increase of CVI after phacoemulsification was found, especially in the luminal vascular component [81,83]. The increased CVI may be associated with the choroidal inflammation induced by surgical trauma or with the intraocular pressure drop after cataract surgery [81,83].

The only report analyzing CVI in macular surgery suggested a significant CVI decrease after macular surgery [82]. The significant reduction in CVI after surgery seems to imply that the choroidal layer is affected by vitreomacular disease.

Mechanical traction over the macular region, either by vitreomacular traction or epiretinal membranes, may lead to choroidal thickening via different pathways. The anteroposterior traction on the retina may affect both the RPE and the choroid, as suggested by Stefansson [84].

Although intriguing, there is no current advantage in performing CVI analysis in surgical patients, as CVI does not predict nor influence preoperative and postoperative patient management.

## 12. CVI in Other Conditions

**Figure 5:**
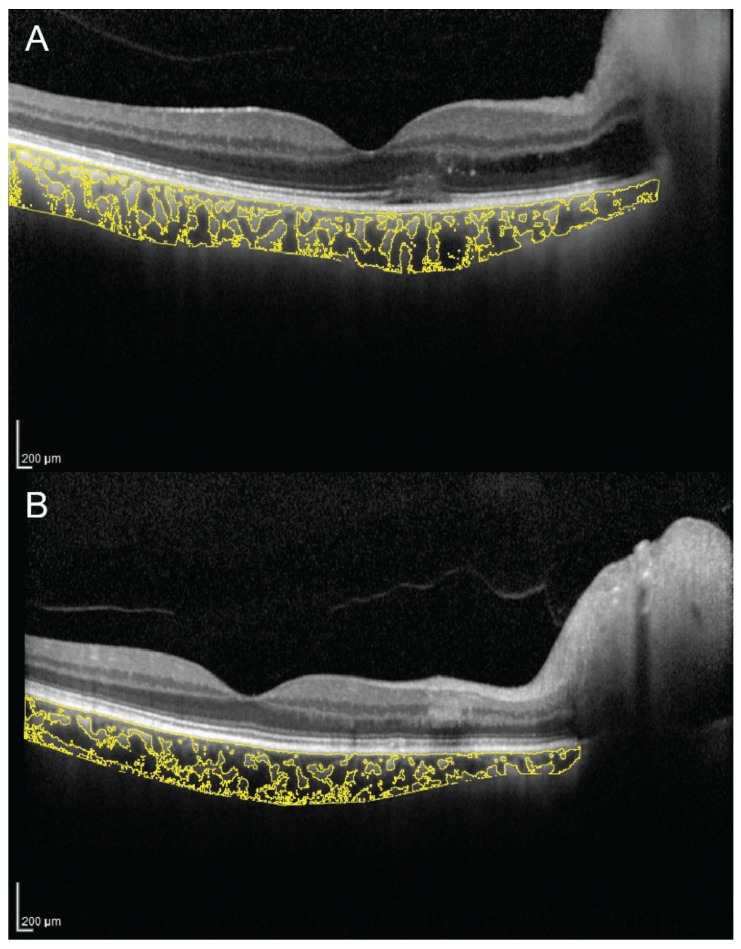
Choroidal vascularity index measurement in a patient with arteritic anterior ischemic optic neuropathy (**A**) and a patient with nonarteritic anterior ischemic optic neuropathy (**B**). (**A**) CVI was 65.1% in the patient with arteritic anterior ischemic optic neuropathy. (**B**) CVI was 68.3% in the patient with nonarteritic anterior ischemic optic neuropathy.

CVI has been evaluated in several other conditions potentially affecting the choroidal circulation. A recent study evaluated macular and peripapillary CVI in patients with arteritic anterior ischemic optic neuropathy (A-AION), nonarteritic anterior ischemic optic neuropathy (NA-AION) and control subjects [85]. Patients with A-AION showed significantly lower macular and peripapillary CVI (Figure 5). In A-AION, the vasculitis of posterior ciliary arteries occurs proximally to their division into paraoptic and choroidal branches, resulting in both optic nerve and choroidal hypoperfusion. CVI may be useful to evaluate quantitatively choroidal ischemia and distinguish A-AION from NA-AION [85].

In contrast with the results of Pellegrini et al., a subsequent study reported a decreased CVI also in NA-AION [86]. Since the study employed a different OCT technique, it is difficult to draw definitive conclusions. However, abnormalities of the choroidal circulation may contribute to the pathogenesis of NA-AION. Another condition in which CVI may have a diagnostic utility is carotid cavernous fistula, an abnormal communication between carotid arterial system and cavernous sinus. The increased venous pressure in eyes with carotid cavernous fistula results in choroidal vascular engorgement, with increased CT and CVI [87].

Hemodialysis for end-stage renal disease causes a sudden change in the amount of body fluids, potentially affecting the choroidal structure. Choroidal changes were investigated immediately before and after hemodialysis. Despite CT having significantly decreased, no change was observed for CVI [88]. Another study compared CVI in smokers and nonsmokers, reporting a lower value in smokers, and a dose-dependent relationship between CVI and pack-years [89]. The association of smoking with vascular dysfunction is well recognized, and CVI may serve as an indicator of systemic vascular health status.

The choroid plays a role in emmetropization and development of the refractive state, and eyes with unilateral amblyopia show increased CT [90]. A recent case-control study documented a higher CVI in amblyopic eyes compared to fellow eyes and control eyes [91]. Moreover, a strong negative correlation between CT and CVI was found in amblyopic eyes, while a positive correlation was found in normal eyes. The authors suggested that decreased blood supply to the outer retina may represent a possible mechanism for amblyopia in anisometropic hyperopia [91].

## 13. Conclusions

Remarkable improvements in OCT technology have resulted in highly sophisticated, noninvasive machines which produce images with quasi-histologic resolution of the retina and choroid [92]. Postproduction automated imaging analysis with public-domain software allows precise quantitative analysis of binarized OCT images. In this regard, CVI is a newly proposed tool which can be used to quantitatively measure and analyze choroidal vascular system. This novel OCT parameter has enabled new directions in research relating to the rules of the choroid in both healthy and pathological eyes.

In the clinical setting, measuring the proportions of the choroidal vasculature components provides a deeper understanding of the choroidal changes occurring in eye diseases. Based on this concept, CVI evaluation is more informative compared to CT measurement alone.

Although this review highlights new insights regarding the choroidal vascularity analysis in normal and in diseased eyes, CVI measurement remains in a state of rapid evolution and development. Since a complete and good visualization of the entire choroid is necessary for both automated and semiautomated CVI analysis, artifacts due to OCT signal attenuation and projection remain major limitations.

Nevertheless, it seems clear even at this early stage that CVI is likely to become an integral tool in clinical practice.

In a near future, CVI analysis could be embedded in the software of all OCT machines, providing additional information about the vascular status of the choroid.
